# Association between Iris Biological Features and Corneal Biomechanics in Myopic Eyes

**DOI:** 10.1155/2021/5866267

**Published:** 2021-11-18

**Authors:** Lin Fu, Qi Dai, Pengzhi Zhu, Xu Jia, Fangjun Bao, Xiaoyu Chen, Yana Fu, Hengli Lian, Weihua Yang, Yufeng Ye

**Affiliations:** ^1^School of Ophthalmology and Optometry, Eye Hospital, Wenzhou Medical University, Wenzhou 325027, China; ^2^Guangdong Medical Devices Quality Surveillance and Test Institute, Guangzhou 510663, China; ^3^Wenzhou Medical University, Wenzhou 325027, China; ^4^Affiliated Eye Hospital, Nanjing Medical University, Nanjing 210029, China

## Abstract

**Purpose:**

Iris biological features such as surface features and profile characteristics reflected the development of iris stroma and microvessels. Iris vessels and microcirculation are still lack of effective detection methods, and we can directly observe only the iris surface biological characteristics. This cross-sectional study evaluated the association between iris surface biological features and corneal biomechanics in young adults with myopia.

**Methods:**

We recruited 152 patients with myopia aged ≥18 years, from the Eye Hospital of Wenzhou Medical University, who had complete Corneal Visualization Scheimpflug Technology (Corvis ST) data and graded iris surface features. Iris surface features included crypts, furrows, and color measured from digital slit lamp images. The biomechanical properties of the cornea were assessed using Corvis ST. Only 1 eye of each participant was randomly selected for analysis. Associations between the iris surface features and corneal biomechanics were analyzed using linear regression models. The grade of iris crypts, furrows, and color and corneal biomechanical parameters measured with Corvis ST was the main outcome measures.

**Results:**

The iris crypts were significantly associated with deflection amplitude at the first applanation (A1 DLA, *β* = 0.001, *P* = 0.013), A1 delta arc length (A1 dArcL) (*β* = −0.001, *P* = 0.01), maximum delta arc length (dArcLM) (*β* = −0.004, *P* = 0.03), and stiffness at the first applanation (SP-A1) (*β* = −2.092, *P* = 0.016). The iris furrows were only associated with integrated radius (*β* = −0.212, *P* = 0.025). Iris color was found not related with corneal biomechanical parameters measured via Corvis ST.

**Conclusions:**

Iris surface features were associated with corneal biomechanical properties in myopic eyes; more iris crypts were associated with lower corneal stiffness while more extensive furrows were related with higher corneal stiffness. Iris crypts and furrows may provide useful information on corneal biomechanical properties in myopic eyes.

## 1. Introduction

Cornea is an important part of the refractive system of the human eye, and its refractive power accounts for more than 3/4 of the whole refractive system. Laser corneal refractive surgery can correct ametropia by cutting the corneal stroma to change the curvature of the anterior surface. Refractive surgery for myopia will reduce the number of central corneal lamellar and change the structure and biomechanical properties of cornea [1]. Different cutting methods and depths have different effects, which may affect the predictability of surgery and the occurrence of complications such as refractive regression and keratectasia [2, 3]. Therefore, the biomechanical properties of the cornea play an important role in the maintenance of the cornea shape and the design of refractive surgery, especially in the diagnosis of some latent corneal diseases such as keratoconus and other keratectasia before refractive surgery. Sufficient evaluation of the corneal biomechanical properties has a strong guiding significance for the safety of refractive surgery.

Noninvasive measures of corneal biomechanics in vivo mainly include electronic speckle pattern interferometry (ESPI), ocular response analyzer (ORA), and Corneal Visualization Scheimpflug Technology (Corvis ST, Oculus, Wetzlar, Germany). [4] However, in China, only a few refractive surgery centers have the equipment to examine the corneal biomechanics. In the absence of such equipments, doctors cannot determine the patient's corneal biomechanical properties initially. Cornea is also part of the outmost layer of the eyeball, and the corneal biomechanics was reported to have relationship with the elasticity of the sclera, lamina cribrosa, and peripapillary ring [4]. So far, as we know, the expansions in the inner ocular tissue, particularly the iris, are less studied. The iris has a variety of biological features such as intuitive surface features and profile morphological characteristics of iris stroma and microvessels. All these characteristics reflect the development of iris stroma and vascular circulation. Iris blood vessels and microcirculation still lack of effective detection methods, and we can directly observe only the iris surface biological characteristics.

A grading system was recently established to evaluate the iris surface features, including iris crypts, furrows, and color that are observable under a slit lamp. [5] However, the role of iris surface features in ocular function and their associations with other ocular tissues are yet to be fully elucidated. Chua et al. [6] speculated that as crypts surrogate iris stroma, iris having more crypts is more compressible. Thus, iris crypts may be related with iris biomechanics. Nevertheless, to date, the human iris biomechanics has not been measured in vivo.

As part of cornea and iris are of mesodermal origin, we postulate that the iris biomechanics is related with corneal biomechanics [7, 8]. No modality is currently available to directly measure iris biomechanics in humans. Exploring the relationship between the corneal biomechanical properties and iris features may improve the safety of refractive surgeries. Iris surface features, which can be examined under a slit lamp, have been suggested to be associated with iris biomechanics. Thus, this study is aimed at exploring the relationships of iris surface features including iris crypts, furrows, and color with corneal biomechanics.

## 2. Methods

### 2.1. Participants

This prospective study recruited Chinese refractive surgery candidates aged older than 18 years. Participants with ocular pathology other than refractive error were excluded. A simple randomization method was used to select the left eye or the right eye based on a randomized number table according to the order the participants that were recruited.

Approval was obtained from the Institutional Review Board (IRB) of Wenzhou Medical University (IRB approval number: 2020-128-K-113), and the study was conducted according to the tenets of the Declaration of Helsinki. Written informed consent was obtained from all participants.

### 2.2. Ocular Examinations

All participants underwent standard detailed ophthalmic examinations including slit lamp examination, manifest refraction measurement (spherical equivalence, [SE]), and best corrected distance visual acuity. All examinations were performed by an experienced ophthalmologist. The patients also underwent corneal tomography with the Pentacam (Oculus, Wetzlar, Germany), anterior chamber depth (ACD) measurement using the IOL-Master (Carl Zeiss Meditec, Jena, Germany), and biomechanical property assessment using the Corvis ST (Oculus, Wetzlar, Germany).

### 2.3. Iris Photography and Grading

Iris photography was performed with reference to the procedure by Sidhartha et al. [5] Briefly, the color iris images of both eyes were taken using a slit lamp digital camera (DC3; Topcon, Tokyo, Japan) at ×16 magnification in a dark room (20 lux) without flash. The images were captured under an illumination of 45° temporally and a brightness of 30% maximum light beam with a width of >20 mm and height of 14 mm.

**Figure 1:**
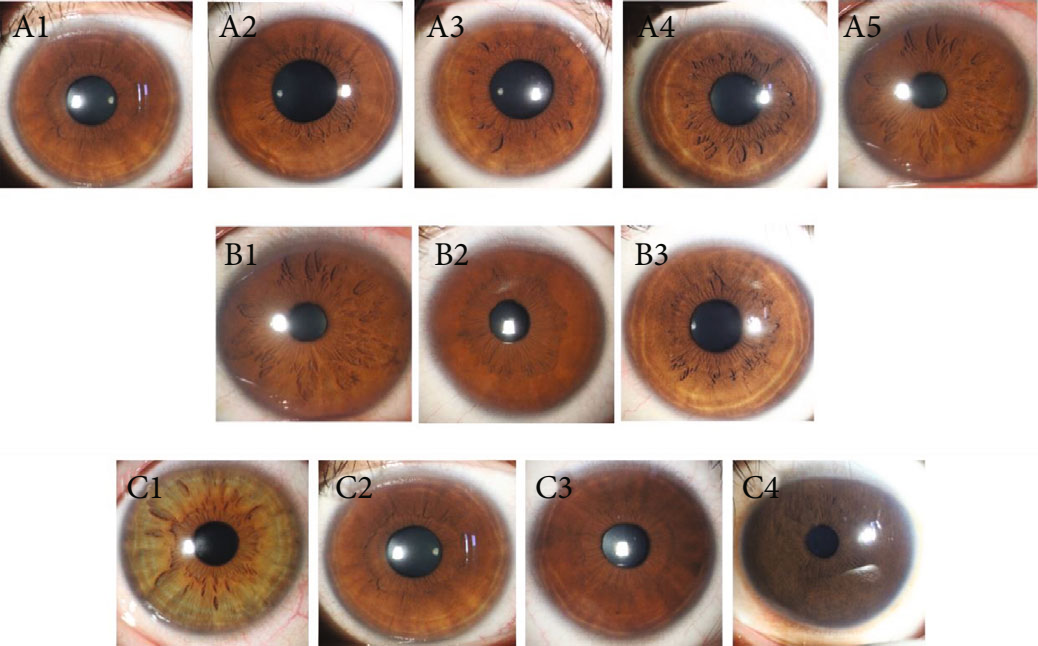
Representative photographs used in grading the iris surface features. A1-A5: grade 1 to grade 5 iris crypts. B1-B3: grade 1 to grade 3 iris furrows. C1 to C4: grade 1 to grade 4 iris color. No grade 5 iris color was observed in this study.

The iris crypts and furrows were graded as follows (Figure 1): iris crypts were categorized into 5 grades as grade 1, no crypts; grade 2, 1-3 crypts, grade 3, at least 4 crypts with a diameter < 1 mm; grade 4, at least 4 crypts with a diameter > 1 mm; and grade 5, numerous crypts with a diameter > 1 mm, nearly covering the whole iris. Meanwhile, furrows were categorized into three grades based on circumferential extent and the number of furrows: grade 1, no furrows; grade 2, less than 5 furrows and the extent was ≤180°; and grade 3, at least 5 furrows present, and the extent was ≥180°. The iris color was also graded according to Sidhartha's criteria in which darker iris was given higher grades [5]. Iris color was divided into 5 grades, but no patient in this study manifested grade 5 iris color.

### 2.4. Corvis ST Measurement

The corneal biomechanical properties were assessed using the Corvis ST following a standard procedure described previously [9]. A rapid air puff was released to the cornea to induce corneal deformation. Dynamic corneal deformation was recorded using a high-speed Scheimpflug camera with full corneal cross-sections. The camera can capture 4330 frames per second and 140 sequential horizontal images with a range of 8.5 mm. The parameters of corneal dynamic response were calculated with a recording measurement time of 30 ms. The biomechanical corrected intraocular pressure (bIOP) was also generated using Corvis ST. The measurements were regarded as reliable when an “OK” quality score was displayed on the device monitor.

The biomechanical parameters measured with Corvis ST recorded were IOP; bIOP; maximum DA (DA max) at the first applanation; time at the highest concavity (HC time); time at the first and second applanation (A1 time and A2 time, respectively); corneal velocity at the first and second applanation (A1 velocity and A2 velocity, respectively); DA; deflection length (DLL); deflection amplitude (DLA) and delta arc length (dArcL) at A1, HC, and A2; PD, radius, and maximum deflection amplitude at the first applanation (DLA max); maximum delta arc length (dArcLM); maximum inverse radius (max inverse radius); maximum DA ratio (DA ratio max) at 2 mm and 1 mm; central corneal thickness (CCT); integrated radius; and SP-A1.

### 2.5. Statistical Analyses

The associations between corneal biomechanical parameters measured with Corvis ST (dependent variable) and iris surface features (independent variable) were assessed using linear regression models. Model 1 was adjusted for age and sex, and model 2 was further adjusted for potential confounders including ACD, CCT, and bIOP. All statistical analyses were performed using SPSS version 23.0 software (SPSS for Windows, IBM Corp., Armonk, NY, USA). A *P* value <0.05 was considered statistically significant.

## 3. Results

After excluding 27 patients due to poor quality of iris photography (*n* = 7), unreliable Corvis measurements (*n* = 9), and IOP higher than 21 mmHg (*n* = 11), 152 patients were included in the final analyses.  Table 1 shows the demographic and ocular characteristics of the participants. The mean age was 25.21 ± 5.93 years, and 39.5% were female. The mean refraction (SE) was −5.75 ± 2.39 D. With respect to the grade of the iris surface features, only 1 eye (0.7%) showed grade 5 iris crypts, and most patients had grade 1 (34.2%) and grade 2 (40.8%) features. The distribution of the iris furrows showed a reverse pattern: only 10 eyes (6.6%) were grade 1, and 103 eyes (67.8%) were grade 3.  Table 2 shows the mean values of the corneal biomechanical parameters measured via Corvis ST.

The association between the iris surface features and corneal biomechanical parameters is shown in Table 3. After controlling the age and sex, the crypt grade was positively correlated with the A1 DLA (*β* = 0.001, *P* = 0.014) and negatively correlated with A1 dArcL (*β* = -0.001, *P* = 0.017). Similarly, a higher iris crypt grade remained significantly associated with larger A1 DLA (*β* = 0.001, *P* = 0.013) and shorter A1 dArcL (*β* = −0.001, *P* = 0.01) after adjusting for additional covariates including ACD, CCT, and bIOP. Moreover, higher iris crypt grades were associated with smaller SP-A1 (*β* = −2.092, *P* = 0.016) which represents corneal stiffness and dArcLM (*β* = −0.004, *P* = 0.03) as well. Iris furrows were only associated with integrated radius after adjustment for ACD, CCT, and bIOP. With the increasing of iris furrows, the integrated radius (*β* = −0.212, *P* = 0.025) decreased. No association was found between the iris color and corneal biomechanical parameters measured via Corvis ST.

## 4. Discussion

The role of iris surface features in ocular function and their associations with other ocular tissues are yet to be clarified. The distribution of iris surface features varies by age, race, and gender [10]. Therefore, in this study, we focus our target population on young myopes who are refractive surgery candidates to see the impact of iris surfaces features on corneal biomechanics and assist preoperative evaluations of corneal status before laser refractive surgeries.

Our study found that iris surface features are associated with corneal biomechanics. Specifically, more iris crypts were associated with larger A1 DLA, shorter A1 dArcL, dArcLM, and reduced SP-A1. Further, more extensive iris furrows were related with smaller integrated radius. Since shorter ArcLM, larger A1 DLA and integrated radius suggest lower level of corneal stiffness [11, 12]. Hence, these findings indicate that eyes with more iris crypts have softer corneas, while more furrows have stiffer cornea. Meanwhile, iris color was not associated with corneal biomechanics. Therefore, the features of iris crypts and furrows may provide useful hints regarding the corneal biomechanical characteristics of myopic eyes.

Iris crypts are result of the hypoplasia or atrophy of the iris stroma typically located at the midcentral area of the iris. Iris furrows are the sites where the iris folded and contracted as protuberant lines on the peripheral iris. Previous literature only supports indirect relationships between iris surface features and iris biomechanics in glaucoma studies [13, 14]. For example, in a finite element study of patients with a history of angle closure glaucoma, iris stiffness was higher in the patients than that in healthy participants [13]. Conversely, the presence of more iris crypts was associated with a thinner iris thickness [5] and a lower rate of acute primary angle closure [15]. Therefore, it is said that in angle closure glaucoma (ACG) eyes, iris crypt may be a protective factor [13].

The reason that there is relationship between iris surfaces features and corneal biomechanics is that the iris stroma and corneal stroma are adjacent ocular tissues and from the same embryonic origin of mesoderma [7, 8]. In addition, ocular tissues are said to be expanded in various patterns, such as posterior pole expansion [16], equatorial expansion [17], and axial expansion [18], in persons with myopia. The iris crypts, iris furrows, and cornea may also be a result of ocular expansion. Moreover, our results showed that iris crypts correlated with deflection parameter A1 DLA but not with deformation parameter of A1 DA. Corneal deflection amplitude is the pure corneal component of the corneal DA minus the whole eye movement. This suggested that corneal deflection, which excludes the influence of eye movement, is more related with iris crypts than corneal DA.

For patients preparing for laser refractive surgery, the most dangerous postoperative complication is the development of keratectasia such as keratoconus [2, 3, 19]. Especially for some patients with high myopia and thin cornea, the risk of keratoconus will be greatly increased by close to the upper limit of corneal cutting, if the patient had abnormal corneal biomechanics before surgery. For doctors without corneal biomechanical examination equipment, even if equipped with Pentacam or some similar equipment, it is sometimes difficult to screen out preclinical keratoconus patients. The results of this study suggest that laser refractive surgeons should pay special attention to corneal biomechanical properties in patients with high levels of crypts and less furrows and reduce the amount of cutting or change the surgical option if no corneal biomechanical measurement conditions are available.

The main limitation of this study is that we only included participants with myopia, and the mean age is 25.21 ± 5.93 years, which is a young population. Our results only represented the associations between the iris surface features and corneal biomechanical properties in persons with myopia as we want to see the role of iris surface features on the refractive surgeries. Further studies should be carried out to also enroll individuals with emmetropic and hyperopic eyes of a wider age range, if it is needed. Another limitation is the cross-sectional study design, which prevented us from evaluating the causal relationship between corneal biomechanics and iris surface features. However, it is difficult to identify a causal relationship between the features. A longitudinal design may help to investigate the dynamic changes of these parameters and enrich understanding of their relationships.

## 5. Conclusion

Iris surface features were associated with the corneal biomechanics measured using Corvis ST. More iris crypts indicated softer corneas while more furrows suggested stiffer corneas in myopic eyes. The iris surface features may aid the assessment of corneal biomechanical properties in persons with myopia.

## Figures and Tables

**Table 1:** Clinicodemographic patient characteristics (*n* = 152).

Characteristics	Value
Age, years	25.21 [5.93]
Sex	
Male	92 (60.5%)
Female	60 (39.5%)
Pupil diameter, mm	3.15 [0.62]
ACD, mm	3.70 [0.25]
Axial length, mm	25.96 [1.07]
bIOP, mmHg	15.38 [1.88]
CCT, *μ*m	540.66 [34.02]
Refraction, SE	-5.75 [2.39]
Iris crypt	
Grade 1	52 (34.2%)
Grade 2	62 (40.8%)
Grade 3	27 (17.8%)
Grade 4	10 (6.6%)
Grade 5	1 (0.7%)
Iris furrow	
Grade 1	10 (6.6%)
Grade 2	39 (25.7%)
Grade 3	103 (67.8%)
Iris color	
Grade 1	4 (2.6%)
Grade 2	28 (18.4%)
Grade 3	109 (71.7%)
Grade 4	11 (7.2%)
Grade 5	0 (0%)

Data are presented as the mean (standard deviation) or as *n* (%). ACD: anterior chamber depth; bIOP: biomechanical corrected intraocular pressure; CCT: central corneal thickness; SE: spherical equivalent of refractive error.

**Table 2:** Mean value of the corneal biomechanical parameters.

Corvis parameters	Mean [SD]
DA max, mm	1.06 [0.10]
A1 time, ms	7.42 [0.23]
A1 velocity, ms	0.15 [0.02]
A2 time, ms	22.02 [0.35]
A2 velocity, ms	-0.28 [0.03]
HC time, ms	16.7 [0.36]
PD, mm	4.99 [0.24]
Radius, mm	6.68 [0.71]
A1 DA, mm	0.15 [0.01]
HC DA, mm	1.06 [0.10]
A2 DA, mm	0.32 [0.06]
A1 DLL, mm	2.24 [0.18]
HC DLL, mm	6.42 [0.42]
A2 DLL, mm	2.71 [0.56]
A1 DLA, mm	0.09 [0.01]
HC DLA, mm	0.93 [0.09]
A2 DLA, mm	0.1 [0.01]
DLA max, mm	0.94 [0.09]
A1 dArcL, mm	-0.02 [0]
HC dArcL, mm	-0.13 [0.02]
A2 dArcL, mm	-0.02 [0]
dArcLM, mm	-0.15 [0.02]
Max inverse radius (mm^−1^)	0.19 [0.02]
DA ratio max, 2 mm	4.32 [0.35]
DA ratio max, 1 mm	1.56 [0.04]
Integrated radius (mm^−1^)	9.11 [0.86]
SP-A1	102.44 [18.39]

SD: standard deviation; DA: deformation amplitude; Max: maximum; A1: the first applanation; A2: the second applanation; HC: highest concavity; PD: peak distance; DLL: deflection length; DLA: deflection amplitude; dArcL: delta arc length; dArcLM: delta arc length max; SP-A1: stiffness parameter at the first applanation.

**Table 3:** Associations between iris surface features and corneal biomechanical parameters.

	Model 1^∗^		Model 2^†^	
	*β*	*P* value	*β*	*P* value
Iris crypt				
A1 DLA	0.001	0.014	0.001	0.013
A1 dArcL	-0.001	0.017	-0.001	0.010
dArcLM	-0.002	0.309	-0.004	0.030
SP-A1	-1.942	0.255	-2.092	0.016
Iris furrows				
Integrated radius	-0.190	0.100	-0.212	0.025

^∗^Model 1 was adjusted for age and sex. †Model 2 was adjusted for age, sex, anterior chamber depth, central corneal thickness, and bIOP. A1: the first applanation; DLA: deflection amplitude; dArcL: delta arc length; dArcLM: delta arc length max; SP-A1: stiffness parameter at the first applanation.

## Data Availability

The datasets used and/or analyzed during the current study are available from the corresponding author on reasonable request.
